# Right ventricle remodelling: from *in vitro* to *in vivo* and from simple to complex models

**DOI:** 10.1016/j.jmccpl.2025.100298

**Published:** 2025-04-14

**Authors:** Paula A. da Costa Martins, Martina Calore, Jordy M.M. Kocken

**Affiliations:** a CARIM School for Cardiovascular Disease, Faculty of Health, Medicine and Life Sciences, Maastricht University, 6229 HX Maastricht, the Netherlands; b Department of Physiology and Cardiothoracic Surgery, University of Porto, 4200-319 Porto, Portugal; c Department of Biology, Universitá degli Studi di Padova, Padova, Italy; d Department of Cardiovascular Sciences, KU Leuven, Leuven, Belgium

## Abstract

Right ventricle failure (RVF) is a debilitating disease with no cure available. While much is known about the failing left ventricle (LV), many mechanisms and signalling pathways of remodelling are different between the two ventricles. Over the past decades, new insights into the mechanisms of the disease have helped in managing disease progression and improving patient comfort. To study RVF both *in vitro* and *in vivo* and even *ex vivo*, relevant experimental models are required to discover new mechanisms and test novel therapeutic approaches. During the past decades many strategies to mimick RV hypertrophy (RVH), to some extent, have been developed and described with using varying methods of disease induction. Such models either require genetic modulation, surgical intervention, chemical injections, or changes in environmental exposure.

As each approach has a different set of requirements of facility and skills, one needs to carefully consider which one better suits a specific study or answer a specific research question. In this review, we provide an overview of the most common *in vitro* techniques, both 2 and 3 dimensional, *in vivo* and promising *ex vivo* approaches to study RV remodelling.

## 1 Introduction

Right ventricle failure (RVF) is reported as the most common cause of death in patients suffering from pulmonary hypertension (PH) [1,2]. PH is defined by a mean pulmonary arterial pressure above 20 mmHg measured by right heart catheterization [3]. With a prevalence of 1 % worldwide and patients requiring multiple hospitalizations during disease progression, PH is an enormous burden on society and health care [4,5]. PH can be divided into five classes, based on its aetiology [3]: Class 1, pulmonary arterial hypertension (PAH) is a rare but progressive narrowing of the pulmonary artery (PA); Class 2 includes PH due to left heart disease and it is the most common cause of PH; Class 3 is characterized by PH due to lung disease, while Class 4 is PH associated with pulmonary artery obstructions and Class 5 considers PH with unclear or multifactorial origin [3]. Whereas current therapies mainly focus on reducing pulmonary and RV dysfunction, no definitive cure is yet available rather than lung or heart-lung transplantation [3], which are, obviously, very limited [6].

**Fig. 1:**
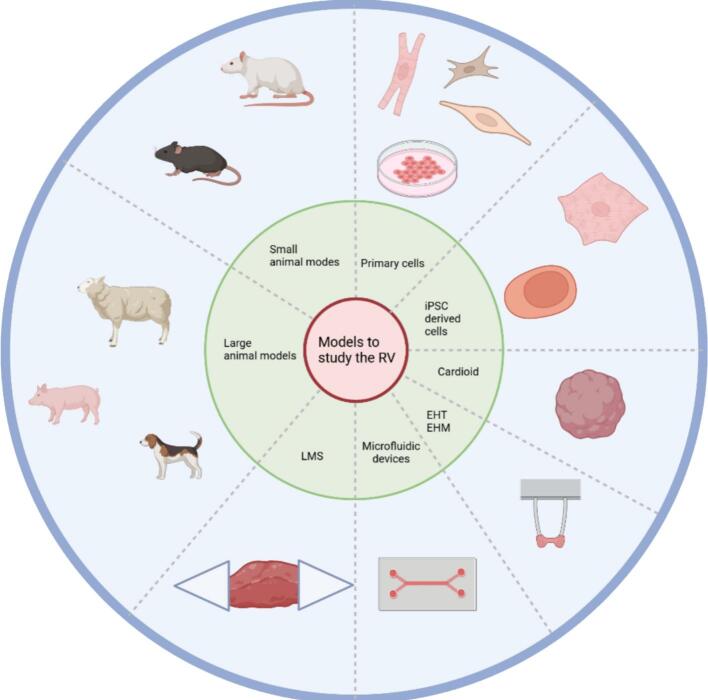
An overview showing the most commonly used models to study RV function. It shows the different two-dimensional cell culture models, followed by three-dimensional cell culture, microfluidic chips, living myocardial slices, and both small and large animal models.

During PH, the RV undergoes hypertrophic remodelling to endure the increased pressure of the pulmonary arteries [7,8]. In an early stage of the disease, remodelling is considered adaptive and it is characterized by increased cardiomyocyte (CM) size, increased angiogenesis and better contractility to maintain cardiac output [9]. When the high pulmonary pressures persist and/or even increase, the RV needs to undergo further remodelling, eventually leading to a maladaptive state, defined by dilation of the RV and reduced cardiac output [3,8,9]. Although much is known about the failing left ventricle (LV), many therapies that work for the LV are not translatable to the failing RV [10]. To further investigate RV remodelling and test potential therapies, experimental models recapitulating RV disease are needed. Here, we provide an overview of the most common *in vitro*, *in vivo,* and *ex vivo* models that are currently used to study morphological, molecular and functional changes in the RV (Fig. 1).

## 2 *In vitro* models

### 2.1 Two-dimensional models

A plethora of different types of *in vitro* models are available, including the traditional two-dimensional (2D) cell culture as well as the more sophisticated three-dimensional (3D) methods, such as engineered (multi) cellular tissues, organoids, and organ-on-chip (OoC). The 2D mono cell culture is still the most commonly used approach, in which a monolayer of one cell type is expanded and treated to induce the remodelling occurring during the RV remodelling [11]. 2D cell culture is relatively cheap when compared to *in vivo* studies. However, these monocellular 2D cultures also come with downsides since they do not mimic complex multicellular tissues, thus presenting limited option for both intercellular interactions and complex extracellular matrix (ECM) interactions, as well as for functional evaluation [11,12]. Another major issue is lack of RV specificity. As, to this date, no RV-specific cell lines are commercially available or have been identified, researchers still rely on freshly isolated RV primary cell lines, which can be limited by the amount of human donor tissues available. Furthermore, it is possible to investigate RV mechanisms in other cell lines and/or validate with more general models or explanted RV tissue. Some of these cell lines are murine CMs, *e.g.* mouse atrial derived HL-1 and rat embryonic ventricular H9C2 cell lines [13]. H9C2 somewhat resemble primary CMs in terms of hypertrophic remodelling, but HL-1 cells, in contrast, lack this hypertrophic response [13,14]. Human CM cell lines that can be used in this context consist on either primary cells that are SV40 antigen-transformed [15,16] or induced pluripotent stem cell derived cardiomyocytes (iPSC-CMs) [17]. Furthermore, with the rise of iPSC research comes increased costs in both material and labour, as chemically defined media for iPSC are more expensive than regular media and iPS cells require more intense maintenance labour. Nevertheless, they are easy to handle, easy to upscale as well as to manipulate, making it possible to mimic different RV cellular conditions like CM hypertrophy, changes in endothelial cell (EC) function, or fibroblast (FB) distress by increased collagen production [11].

A strong advantage of iPSC-CMs is that they can be generated from somatic cells of patients harbouring a genetic mutation, and subsequently, the isogenic control can be obtained by site-specific genome editing using CRISPS/Cas9-based approaches, thus preserving the patient's genetic background [[18], [19], [20], [21]]. Although the introduction of iPSC-CMs has been a step forward into drug discovery and screening tools [22,23], the technique, unfortunately, has some limitations. One is the immaturity of the differentiated cells, as they represent a more foetal-like cell [24]. This is reflected in terms of shape, metabolic and electrophysiological characteristics [25]. During the last decade, many methods have been tested to mature iPSC-CMs, *e.g.*, prolonging time of culture, electrical or chemical stimulation, co-culture with different cardiac cell types, and ECM modulation [6,25,26]. Despite improvement in CM maturity, cells still fail to reach a complete ‘adult-like’ state. [25]. Another downside of using iPSC-CMs is the lack of differentiation towards RV specific lineages. While some studies have shown that it is possible to push differentiation towards atrial or ventricular CMs by addition of retinoic acid [27], no protocol succeeded to generate either LV or RV CMs. On the other hand, heart field specific lineage generation from human embryonic stem cells (ESCs) holds promises in this context [28]. During cardiac development, the LV and RV myocardium have different origins, namely the primary heart field for the LV and the anterior secondary heart field for the RV [29]. The secondary heart field is characterized by expression of *Tbx1* [30], *Isl1* [31], and *Nkx2.5* [32] and is regulated by fibroblast growth factor (FGF)/Wnt signalling [28].

### 2.2 Three-dimensional models

In turn, 3D cultures have been steadily receiving more attention as studies show that culturing cells in 3D impacts their phenotype, making them both better resemble the physiological situation, compared to 2D culture, and increase the maturity of iPSC-CMs [11,12,[33], [34], [35]]. 3D cell culture is generally defined by the ability of cells to grow in all directions, in contrast to the 2D monolayer system, thus better mimicking the complex 3D architecture of the heart [36]. Known examples are engineered heart tissues (EHT) [37], engineered human myocardium (EHM) [38], or cardiac organoids [39] also referred to as cardioids [40] or cardiac microtissues (MT) [41]. Based on human iPSCs or ESCs, these complex cultures can be made either by mixing different cellular populations and casting them into an ECM hydrogel [37,38] or by self-organisation, meaning that the seeded cells autonomously form a structure without external stimuli [39,40].

By using the EHM/ETH model, it is possible to create complex multicellular patches that can easily be modulated *via* genetic editing or chemical stimulation. The ECM can be modified to consist of different types of structural protein like gelatin or collagen, but also with addition of arginylglycylaspartic acid (RGD) binding integrin to improve cell adhesion and reduce apoptosis [42,43]. This leads to increased maturation of iPSC-CMs as revealed by single-cell RNA sequencing [44]. Usage of an EHT/EHM system also has the ability to analyse not only molecular or histological parameters, but also the possibility of assessing complex physiological differences, such as tissue contraction and relaxation, that better resemble the human adult electrophysiological situation than the traditional 2D iPSC cell types [45]. Yet, while these 3D constructs are closer to the human adult tissue, there are still differences between 3D engineered and adult human tissues. As during human development many properties, like circulatory and cellular composition change [46], it is difficult to replicate these changes in an *in vitro* setting and maintain viable cultures. Furthermore, just like with 2D iPSC culture, there are drawbacks as many studies rely on only a few and/or patient-specific iPSC clones, increasing variability between samples, and potentially attributing observed phenotypes to a disease mechanism, while it might be a clone-specific phenotype [47].

Currently, one study has created an EHT using material derived from a patient harbouring a *BMPR2* mutation together with an isogenic control [48]. While the model is not labelled as RV-specific, a contractile difference was observed in the *BMPR2* mutant-derived EHTs, a known RV disease causing mutation as it impairs the hypertrophic response of the RV during adverse remodelling of the pulmonary artery [49], showing that this technique can be used to study RV phenotypes since it recapitulate the patient's phenotype.

### 2.3 Organoid models

Another 3D model is the cardioid, a class of 3D culture, that, being derived from iPSC- or ESC- derived cardiac cells, is characterized by self-assembly in more complex tissue-like structures [50]. Cardioids can be created by either differentiating stem cells directly *in vitro* [39] or by seeding previously differentiated cells followed by stimulation [41]. Some early models have been published that show great potential as a tool for cardiac research due to the relatively low number of cells needed to start with and, in later stages, reduced variability [51]. This is relevant when thinking of upscaling towards high-throughput screenings (HTS) for therapeutic testing [52]. A recently published example shows the creation of a MT similar to the formation of cardioids. A mixture of 5000 cells, 70 % iPSC-CMs, 15 % iPSC-ECs, 15 % iPSC-FBs, was seeded and self-organized into complex 3D structures [41]. This mixture lead to increased maturation of iPSC-CMs due to so called tri-cellular crosstalk but also showed the role of non-CMs in disease as healthy CMs would become arrythmic if cultured with FBs derived from an arrythmogenic patient. Recently, a study in Cell shows to have created a multichambered cardioid including a second heart field derived RV [53]. The authors hypothesised that inhibiting TGF-β and Wnt signalling would lead to formation of a subpopulation resembling the anterior second heart field which, during development leads to RV formation. Together with the earlier mentioned study by Yang et al. [28], pushing pluripotent stem cells into heart field specific lineages might be the key to creating chamber-specific models and subsequently help reduce the need for *in vivo* models to investigate RV remodelling.

### 2.4 Organ on chip models

A more recently developed model is the heart-on-a-chip. OoCs are a small collection of cells cultured in a microfluidic device, a small apparatus using channels to promote constant fluid flow [54]. Earlier iterations were used as small lab-on-a-chip to detect, for example, bacteria [55], changes in pH [56], or even in enzymatic kinetic studies [57]. The first OoC to be described was a lung-on-a-chip, in 2010, by Huh *et al* [58]. This ‘breathing’ OoC was novel due to the fact that it used three distinct channels that were separated by a porous membrane. This was just the beginning of a new era where scientists are trying to recreate an OoC for each organ with the hope that one day connection of multiple chips will allow to investigate whole-body physiology [59]. Heart OoC models are a combination of different cell types and not always of human origin. Some studies used animal-derived cells, while others tried to create an OoC with iPSC- or ESC-derived cells only [60]. However, while they are able to create impressive looking vascularized 3D cultures, they retain a common problem with other models: a lack of chamber specificity and other challenges of using iPSC-CMs not specifically addressed by the 3D culture [61].

## 3 *In vivo* models

While *in vitro* models can help understanding novel pathways or mechanisms, their simplicity still requires *in vivo* validation and remain necessary for pharmacokinetics studies. Although *in vivo* models can validate information observed in patients or *in vitro* models, they are not without challenges and therefore, their use and the information they provide, should be considered with care.

### 3.1 Murine models to study the RV

For the past 100 years, the house mouse (*Mus musculus*) has been one of the most commonly used animals for experimental research due to the fact that mice generate large litters, have a short gestation time, are easy to handle [62]. This, together with the great overlap between human and murine genomes, makes them ideal animals for studying disease phenotypes or design new genetic models [63]. Besides mice, rats are also very frequently used due to their availability and better access to tools for extensive cardiac functional characterization. While larger animal models overlap better with human physiology, their genome is not always fully annotated, making a translational step much more challenging [64]. When investigating genetic influences on the RV function, mouse [65] and, to a lesser extent, rats [66] are ideal candidates due to their short gestation times and big litter sizes, reducing the time and costs it takes for creating genetically modified animals. Furthermore, it is possible to induce PH by changing physiological parameters, *e.g.* through exposure to chronic hypoxia [67] or chemical stimulus (*e.g.* monocrotaline) [68]. Surgical models (*e.g.* pulmonary artery banding) [69] have been developed over time as well, together with spontaneous or idiopathic generation of PH (*e.g.* the fawn-hooded rat) [70]. It is also possible to mix different types of induction as is seen with the Sugen-hypoxia (SuHx) model [71]. Genetic models allow the evaluation of the effect of mutations found in patients with PH both cellular and tissue level and the ability to test possible treatments and potentially find novel mechanisms of the disease.

Physiological induction of PH in rodents can be done with exposure to chronic hypoxia for prolonged periods. While this is a relatively easy method, mice only develop a mild phenotype, recovering towards an healthy state after returning to normoxia [72]. This is somewhat harsher to rats, who develop a more severe phenotype, which is still considered milder when compared to humans upon chronic exposure to hypoxia [73]. One way to exacerbate the phenotypes in rodents is to combine hypoxic conditions with the VEGFR2 antagonist, Sugen 5416, a tyrosine kinase inhibitor. Sugen was first described in 1999, by Fong et al., as a potential anticancer drug directly targeting VEGFR2 and leading to endothelial cell dysfunction and apoptosis [74]. SuHx models rely on weekly injections of Sugen 5416 and three weeks of hypoxia to develop angio-obliterative lesions, similar to the so-called “plexiform” lesions that are observed in human PAH patients [71].

An alternative model uses monocrotaline (MCT), a chemical compound isolated from *Crotalaria* genus seeds that when injected in rats is metabolized by the liver into monocrotaline pyrrole [75]. This metabolite, then causes inflammation of the PA, resulting in a PAH phenotype that progressively worsens over time [76]. MCT metabolites also cause inflammation in the lungs, kidneys, and liver, leading to co-morbidities that might influence the survival outcome of the animals [75]. An interesting study by Kawade et al. also pointed an age-related survival rate of MCT in rats, showing that young rats injected with MCT are prone to develop a similar phenotype as adult rats, yet pass away at earlier and variable times [77]. This reveals one of the challenges of using MCT, namely, its variability within animals. Why variability within the MCT model persists is not yet elucidated, however it might be related to differences in immunological response post injection [78]. While MCT has its downsides like increased mortality or organ toxicity, a major advantage is that a single intraperitoneal injection will suffice to induce PAH.

Another method used to induce PH in rodents is by surgical intervention. Examples of this are pulmonary artery banding (PAB) [79] or left pneumonectomy, sometimes also in combination with Sugen 5416 [80]. PAB was introduced in 1952 as an palliative surgery technique to treat excessive pulmonary flow in young patients suffering from congenital cardiac defects [81]. In rodents, PAB leads to increased mean pulmonary arterial pressure and subsequent RV remodelling to maintain proper cardiac flow. During PAB, a suture or clip is placed around the PA and then closed to create an acute pressure overload and subsequent RV adaption, as the size of the suture or clip determines PA narrowing and subsequent remodelling [82]. PAB also allows for investigating RV adaptations independent of PA adaptations, which can be seen as either an advantage or disadvantage. This is in contrast to MCT and hypoxia, which affect both pulmonary and systemic circulation. Pneumonectomy, in turn, consists of disconnecting the left branch of the PA, which, by creating a volume overload in the right branch mimics volume overflow in the lung. When combined with Sugen 5416, rats will develop a similar phenotype as the before mentioned SuHx model, suggesting that hypoxia is not needed as a second hit for Sugen to induce PA phenotypes [82]. While surgical models can help developing a more consistent phenotype within an experimental group, they require surgical specialists and surgical equipment on top of the standard animal housing.

A known model of idiopathic PH development, is the fawn-hooded rat (FHR) which spontaneously develops PH with phenotypes increasing in severity when exposed to mild chronic hypoxia [83]. The FHR carries a mutation in both the red-eyed dilution gene leading to a platelet storage deficiency [84] and in Bpfh-1 which is known to lead to the systemic increase in blood pressure [85]. The FHR starts to develop PH roughly ten weeks after birth and when subsequently exposed to four weeks of hypoxia a severe phenotype will develop around the age of 14 weeks [83]. A study with hyperbaric treatment to improve oxygen uptake has shown to reduce progression of the phenotype, but no reversal of the hypoxia induced remodelling as seen in mice undergoing hypoxia exposure followed by a return to normoxia [86].

### 3.2 Large animal models to study the RV

Alongside small animal models, larger animals such as pigs [87,88] or to a lesser extent sheep [89] are used to elucidate new mechanisms of RV failure. As pigs are more similar to humans in terms of size, weight, and cardiac function, they are considered an ideal animal for toxicity and physiological studies [90]. However, they require more specialized housing, equipment, and staff for both caretaking and follow-up studies [91]. The current pig models of RV remodelling vary among different surgical techniques such as PAB [92] and valve replacement mimicking treated Tetralogy of Fallot (ToF) [87]. ToF is the most common congenital heart defect and is characterized by pulmonary stenosis, ventricular septum defects, and RV hypertrophy leading to cyanosis [93]. Current treatments focus on surgical repair or pharmacological treatment of periods of cyanosis [94]. Another form of PH studied using large animal models is chronic thromboembolic pulmonary hypertension (CTEPH). CTEPH is a type of PH that originates after a pulmonary embolism and characterized by a combination of defective angiogenesis with endothelial dysfunction and impaired fibrinolysis [95]. To study CTEPH, animals will receive injections with exogenous materials like enbucrilate tissue adhesive after left PA ligation [96] or injection with polystyrene microspheres [97] to mimic the pathophysiology [96]. While the CTEPH models closely resemble the RV adaptation observed in patients, the induction does not resemble the associated pulmonary disease at all and therefore cannot be used to study chronic embolism aetiology. Attempts were made with both canine [98] and murine [99] models concerning blood clots to favour the pathophysiology of the disease better; however, these models showed problems, such as lack of phenotype and revealed clot lysis complications as some animals underwent cloth lysis independent of treatment [[98], [99], [100]].

While even more animal models have been described, the ones mentioned earlier are the most used, with varying degrees of complexity in both disease induction as general care. Table 1 provides an overview of the animal models that are less commonly used to study RV, but are still valuable depending on the research question into different aetiologies of PH.

**Table 1:** An overview of lesser used and more complex *in vivo* models to study the failing RV.

Animal	Method	RV phenotype	Reference
Rat	Abdominal aortacaval shunt + MCT	Variable RV decompensation times (possibly due to MCT)	[101,102]
Rat	Abdominal aortacaval shunt	RV phenotype mimics shunt-related PH	[103]
Piglet	Abdominal aortacaval shunt	Model of CTEPH	[104]
Sheep	Aortopulmonary shunt	Mild dysfunction, but matches pediatric patients after shunt	[[105], [106], [107]]
Dog	PAB + shunt	Range of RV dysfunction possible based on surgery	[108]
Pig	PAB + pulmonary regurgitation	Tetralogy of Fallot pathophysiology	[87]

While they play an important role in disease, genetic mutations are rarely single-hit mechanisms and therefore are usually combined with other methods of PH induction to induce the RV remodelling [72]. Together with the rise of iPSC-CMs and subsequent development of 3D cell cultures and OoC, it might be possible that there will be a societal push to replace genetic *in vivo* models with patient-derived genetic models due to their availability to validate mechanisms and limit animal studies for pharmacokinetic studies prior to clinical trials, as there is a request to reduce the number of animals used in science.

Another challenge of animal science is the lack of translational value, as patient phenotypes due to a specific mutation might not be well recapitulated in rodents harbouring the same mutation [109].

## 4 *Ex vivo* models

### 4.1 Living myocardial slices

Recently, a novel technique has emerged to further cardiac research that might lead to new insights in how the RV functions and can be influenced towards better function. Living myocardial slices (LMS) are used to generate 100-to-400-μm-thick slices of living myocardial tissue that are placed in a small biomimetic chamber for prolonged culture [110]. This has been done with both human and animal derived cardiac tissues, underlining this technique is not limited to only one species. The first mention of this technique was in 1933 by Pincus, who studied oxygen consumption in different excised rat tissues, including cardiac ventricular tissue [111]. However, the method, as we know today, started to regain traction around 2017 when Watson et al. published a novel approach to isolate and culture these LMS [112]. Since then, the yearly number of publications using this method has only increased, leading to expanded knowledge on setup and maintenance of the LMS [113,114]. Not only does LMS maintain the cellular complexity of the donor tissue, but it is also possible to pace the slices to contract, making them an ideal subject for both pharmacological and electrophysiological studies, and afterwards freeze or fix the slices to for perform molecular or histological studies respectively [115]. Some groups have already shown the possibility of making adjustments to the pacing program to induce arrhythmias in the slices [116] or increased stretching to mimic mechanical overload on the LMS [117]. Recent publications have shown the possibility to produce LMS from rat [117], pigs [114], and human material [114,116] without major drawbacks. While it is possible to use either atria or ventricle tissue, until this day, no study using RV tissue has yet been published.

Compared to EHT/EHM and cardioid models, LMS retain increased maturation of cells and a cellular composition closer to the physiological situation, while also including increased heterogeneity due to of different genetic backgrounds of the tissue donors. Depending on the research question, one should be conscious whether they should go the EHT/EHM or cardioid models or LMS. The LMS system has advantages over other techniques as it maintains cellular complexity as a whole tissue, allows the use of patient material without the need for extensive culture and processing, and enables analysis of additional parameters such as RV-secreted factors [118]. Specifically, these factors can be helpful in elucidating whether the (mal)adaptive RV has any influence on the PA through cytokines or extracellular vesicles (EVs). Conditioned media from these slices can be used on patient derived pulmonary artery endothelial cells (PAECs) to assess a potential functional change upon treatment. However, while access to animals and their subsequent RV tissue comes with challenges, access to patient material is very limited due to scarce number of donors [119], ethical regulation [120], and the need for even more specialized centres to collaborate and acquire these tissues [121]. Furthermore, while dedifferentiation of CMs is reduced due to mechanical stimuli, the process is still present and can influence long term read outs [122].

Yet, a small number of studies have successfully studied the effects of Empagliflozin [123] or anti-fibrotic compounds [124] on LMS generated from cardiac tissue retrieved from discarded hearts after transplantation. Addition of Empagliflozin increased the total duration of contraction compared to vehicle treated samples. The aim to define the effect of Empagliflozin on the cardiac tissue without influence of systemic effects shows the advantage of using the LMS system [123]. Another study screened for anti-fibrotic compounds in human fibroblasts and then tested the cardiotoxicity of selected compounds in both iPSC-CMs and LMS [124]. Furthermore, using dissected atrial tissues, another study showed it is possible to evaluate patient material under different pacing modalities, generating a new model to test anti-arrhythmic therapies or investigate mechanisms involved with arrhythmias [116].

## 5 Concluding remarks

“All models are wrong, but some are useful” is a commonly used aphorism coined by George Box in 1976. This was written to describe the lack of complexity of statistical tests, but highlighting that even with these shortcomings, these tests can be beneficial [125]. Yet, this statement can also be used within the earlier discussed RV setting. None of the above-mentioned models are perfect, however, depending on the defined research question, some are good enough to provide meaningfull answers. Table 2 provides an overview of the models described in this review and their advantages and challenges.

**Table 2:** An overview of all listed models to study the RV and their advantages and challenges.

Model	Advantages	Challenges	
*In vitro* models			
Primary human cell culture	Correct phenotype Easy to manipulate Maintain patient genetic background RV specific	Variability between donors requiring more donors to achieve reproducible results Short term culture (for cardiomyocytes) Contamination from other cell sources depending on isolation method	
Commercially available cell culture	Correct phenotype Easy to manipulate Relatively cheap RV specific	Single cell source Variability between donors Scarce availability Short term culture (for cardiomyocytes)	
iPSC derived 2D models	Human models Maintain genetic background of patient Potential to form multiple cell lineages.	Higher costs compared to primary culture Clone variability Remain immature phenotype Single-patient specific observations possible High variability between lines and laboratories Not specific for RV	
iPSC derived 3D models	Human models Maintain genetic background of patient Complex 3D tissue composition More maturation occurs compared to 2D models	Even higher costs compared to 2D iPSC culture Not as mature as native tissues Single-patient specific observations possible High variability between lines and laboratories Not specific for RV	
Organoids	Self-organizing Low number of cells needed Lower variability	Requires vascularization to sustain longer periods	
Organ on a chip	Perfusion possible Combination of chips to mimic multiple organs	Not RV specific yet Not always pure human cells	
Murine *in vivo* models			**PH class (if any)**
Genetic models	Mice and human genome similarity (>90 % synteny conservation) Easy to manipulate, big litters	Physiological data not always comparable between mouse and human Selective breeding requires inbreeding with WT animals every two or three generations to circumvent genetic adaptation Potential compensation mechanisms preventing genetic mutations to show phenotype similar to human disease	Class 1
Hypoxia	Easy to perform	Systemic effect Mild remodelling Reverse remodelling after returning to normoxia	Class 3
MCT injection	Easy to perform Forms plexiform lesions in PA like in human patients with PAH	High variability between groups High mortality in young rats Reportedly does not work in mice	Class 1
Sugen-Hypoxia	Easy to perform Less variability compared to MCT	Requires weekly injections combined with chronic hypoxia	Class 3
PAB	Low variability between operated animals Only affects RV	Acute pressure overload Severity depending on degree of banding and strain of animals	No specific class
Pneunectomy	Similar phenotype to Sugen-Hypoxia model without need for hypoxia	Needs to be combined with Sugen injections	Class 3
Pig *in vivo* models			
Valve replacement	Mimics Tetralogy of Fallot	Requires specialized housing Higher costs than murine models	Class 1
Exogenous material injections	Mimics CTEPH patient phenotype	Cannot be used to study aetiology of CTEPH	Class 4
*Ex vivo* models			
LMS	Maintains 3D composition and complexity Maintains phenotype Maintain patient genetic background Been used on both human and animal tissue Possible to investigate cardiac specific effects RV specific	Dedifferentiation of CMs over time High variability between donors High variability between slices from single donor Lack of healthy human tissue	

With this review we aimed to provide an overview of the most commonly used experimental cardiac models to investigate RV remodelling. However, more complex models exists and can be found in Table 1, but were not discussed in depth because as they were not described extensively in literature. In the near future, the most urgent problems cardiac research should strive to resolve need to include improvements of the current iPSC-derived *in vitro* models towards chamber specificity and in particular, the RV. Two studies show that pushing iPSC lineages into specific heart fields, by inhibiting Wnt and TGF-β signalling pathways during differentiation, managed to create a more RV specific tisse [28,53]. However, at the moment of writing this review there are only two studies showing this technique. Furthermore, if patient material is available, setting up a LMS system might increase translational ability of preclinical studies as adult human material can be used to verify pathways or novel therapies, and also potentially reducing the need for large animal studies. While the RV has often been called the forgotten ventricle [127] the possibility of using patient-derived material, either through iPSC or LMS systems, opens many opportunities to help unravel the unique mechanisms of the remodelling RV. With differences in both response to stress between the failing LV and RV [10], novel therapies are necessary that are RV specific to treat the failing RV and reduce patient mortality.

## CRediT authorship contribution statement

**Paula A. da Costa Martins:** Writing – review & editing, Writing – original draft, Validation, Supervision, Resources, Project administration, Investigation, Funding acquisition, Conceptualization. **Martina Calore:** Writing – review & editing, Supervision, Resources, Project administration, Funding acquisition. **Jordy M.M. Kocken:** Writing – review & editing, Writing – original draft, Investigation, Conceptualization, Formal analysis, Methodology, Project administration, Resources.

## Declaration of competing interest

The authors declare that they have no known competing financial interests or personal relationships that could have appeared to influence the work reported in this paper.
